# Salvage radiotherapy with or without hormonal therapy for biochemical recurrence after radical prostatectomy: A systematic review and meta-analysis

**DOI:** 10.1038/s41391-026-01120-9

**Published:** 2026-05-18

**Authors:** Fortuna Migliaccio, Fabiano Flauto, Chiara Barraco, Giuseppe Neola, Sarah Scagliarini, Ciro Franzese, Emanuele Chioccola, Gaetano Facchini, Roberto Bianco, Alberto Servetto, Luigi Formisano

**Affiliations:** 1 Department of Medicine and Surgery, Federico II University, Naples, Italy; 2 Oncology Complex Unit, Santa Maria delle Grazie Hospital, ASL NA2 NORD, 80078 Pozzuoli, Italy; 3 Translational & Clinical Research Institute, Centre for Cancer, Newcastle University, Newcastle Upon Tyne, UK; 4 UOC di Oncologia, Azienda Ospedaliera di Rilievo Nazionale Cardarelli di Napoli, Naples, Italy; 5 Department of Radiotherapy and Radiosurgery, Humanitas Clinical and Research Center - IRCCS, Rozzano, Milan, Italy; 6 Department of Biomedical Sciences, Humanitas University, Pieve Emanuele - Milan, Milan, Italy; 7 Department of Cardiovascular Sciences, Diagnostic Imaging and Time-Dependent Network of Cardiovascular Emergencies, University Hospital “Federico II”, 80128 Naples, Italy

**Keywords:** Cancer therapy, Prostate cancer

## Abstract

**Background:**

Salvage radiotherapy (SRT) is the standard of care for patients with biochemical recurrence (BCR) after radical prostatectomy (RP). Several randomized trials have evaluated the addition of hormonal therapy (HT), including androgen deprivation therapy (ADT) and androgen receptor pathway inhibitors (ARPIs), to SRT; however, differences in patient selection, treatment duration, and endpoints have led to inconsistent results. We performed an updated systematic review and meta-analysis to clarify the clinical benefit of adding HT to SRT in this setting.

**Methods:**

A comprehensive literature search of PubMed/MEDLINE, EMBASE, the Cochrane Library, ClinicalTrials.gov, and ASCO and ESMO meeting abstracts was conducted through April 2025. Randomized phase II-III trials enrolling patients with confirmed BCR after RP and comparing SRT with or without HT were included. Five trials (GETUG-AFU 16, RTOG 9601, RTOG 0534/SPPORT, RADICALS-HD, and SALV-ENZA), comprising 4536 patients, met the eligibility criteria. Hazard ratios (HRs) and 95% confidence intervals (CIs) for metastasis-free survival (MFS), progression-free survival (PFS), biochemical progression-free survival (bPFS), and overall survival (OS) were pooled using fixed- or random-effects models according to heterogeneity. Analyses were stratified by HT duration (short-term vs. long-term), and subgroup analyses were performed based on pathological features.

**Results:**

Short-term HT combined with SRT significantly improved bPFS (HR = 0.57; 95% CI: 0.46–0.71, *p* < 0.00001), PFS (HR = 0.58; 95% CI: 0.49–0.69; *p* < 0.00001), and MFS (HR = 0.82; 95% CI: 0.69–0.96; *p* = 0.02). Long-term HT was also associated with improved MFS (HR = 0.76; 95% CI: 0.61–0.94; *p* = 0.01). No statistically significant OS benefit was observed with either short-term or long-term HT. Subgroup analyses showed a significant MFS benefit in patients with positive surgical margins (HR = 0.68; 95% CI: 0.47–0.98; *p* = 0.04), whereas no clear benefit was detected in patients with Gleason score ≥8 or negative margins.

**Conclusions:**

The addition of HT to SRT improves disease control outcomes in patients with BCR after RP, particularly in those with positive surgical margins, although no overall survival benefit was demonstrated. These findings support a tailored approach to treatment intensification in the salvage setting.

## Introduction

Biochemical recurrence (BCR) after radical prostatectomy (RP) is defined as prostate- specific antigen (PSA) rising ≥0.2 ng/mL, on 2 separate assessments without evidence of clinical or radiological relapse of disease [1]. Indeed, PSA levels are expected to become undetectable after RP. Therefore, PSA testing remains the only validated biomarker for the early detection of residual or recurrent disease [1]. BCR represents a clinically relevant condition, occurring in up to 30–50% of patients undergoing RP as curative treatment [2]. Notably, it may serve as a potential harbinger of disease progression and is associated with an increased risk of distant metastases (DM) and higher prostate cancer-specific mortality (PCSM) [2]. Salvage radiotherapy (SRT) to the prostate bed is widely considered the standard of care for patients with BCR and no radiological evidence of disease relapse after RP [1, 3–7]. Its curative potential, supported by several randomized clinical trials (RCTs), is endorsed by international guidelines [1, 3–7]. Given the role of androgen receptor (AR) signaling in regulating the cellular response to DNA damage [8–10], several RCTs have demonstrated that the synergistic combination of androgen deprivation therapy (ADT) or androgen receptor pathway inhibitor (ARPI) plus SRT improves progression-free survival (PFS), biochemical progression free survival (bPFS) and metastasis-free survival (MFS) compared to SRT alone [11–17]. However, these trials differ significantly in terms of patients’ selection criteria, and therapeutic regimes, and definition of outcomes assessed. Therefore, the benefit of adding hormonal therapy (HT) to SRT remains controversial. Herein, we conducted a systematic review and meta-analysis to provide the most comprehensive and up-to-date synthesis of randomized evidence evaluating the impact of adding HT to SRT, incorporating the most recent data on ARPIs. We aimed to assess all relevant outcomes, including MFS, PFS, bPFS and overall survival (OS) with stratification by HT duration.

## Methods

The protocol for this systematic review and meta-analysis has been published in the PROSPERO database (CRD420251031518). The systematic review was conducted according to Preferred Reporting Items for Systematic Reviews and Meta-analyses (PRISMA) guidelines [18].

### Search strategy

A comprehensive search of records published in PubMed/MEDLINE, EMBASE, the Cochrane Library, ClinicalTrials.gov, ASCO and ESMO meeting abstracts up to April 2025 was carried out. We considered only articles published in the English language and phase III or phase II RCTs including patients with BCR after RP. The MeSH terms used to search in these databases were: prostate cancer AND biochemical recurrence AND androgen therapy OR endocrine therapy AND radical prostatectomy AND salvage radiotherapy.

### Eligibility criteria

The inclusion criteria used to select the RCTs for the final analysis were (1): patients with BCR after RP without clinical or radiological evidence of disease relapse; (2) patients older than 18 years of age (3); phase II or III prospective RCTs comparing SRT with or without ADT or ARPI; (4) RCTs reporting at least one of the following outcomes: MFS, defined as time from randomization to documented metastasis or all cause death; OS, defined as the length of time from randomization to death from any cause or censoring at date of last follow-up; PFS, defined as time from randomization to documented radiological recurrence and/or clinical progression; bPFS, defined as PSA increase after achieving an undetectable PSA after RP as curative treatment. The exclusion criteria were: (1) RCTs including patients who did not receive previous RP; (2) RCTs including patients previously treated with radiotherapy alone or combined with ADT for localized prostate cancer; (3) RCTs including patients who received neoadjuvant ADT; (4) RCTs including ADT in both arms; (5) Non- randomized clinical trials, observational studies, retrospective reports, single-arm trials, case reports/series, duplicate publications.

### Data extraction

A systematic review was conducted by two authors (FM and CB) independently. The software “Rayyan” was used to assess the suitability of the screened papers for inclusion [19]. Any discrepancies were resolved by consulting a third senior author (LF). After removing duplicates, we screened the remaining records by browsing titles and abstracts. After

exclusion of the ineligible records, we analysed the full text of potentially articles and reviewed them for eligibility. Data extracted for each eligible RCTs included: trial name, year of publication, trial phase, number of enrolled patients in each arms, the percentage of patients who received even radiotherapy on pelvic nodes, the duration of ADT (6 or 24 months), and hazard ratios (HRs) for MFS, PFS, OS and bPFS with their relative 95% confidence interval (CIs). We reported all this data collection in Supplementary Table 2.

### Statistical methods

We extracted HRs for PFS, MFS, OS and bPFS, along with their respective CIs from each five studies. When more than one result is available, we consider those with the longest follow-up. In order to minimize a potential bias related to HT duration (6 or 24 months), we categorized the studies in two different groups: short-course HT and long-course HT. The analyses were conducted separately for each group.

### Data synthesis

Data were collected in an electronic database. Meta-analysis was performed using Review Manager software (version 5.4.1) [20]. We used the inverse variance and log (HRs) for pool estimates. Statistical heterogeneity between the trials was tested using Cochrane’s Q statistic and inconsistency was assessed using the I² statistic [21, 22]. A fixed-effects model was applied when heterogeneity was low to moderate (I² ≤ 50%), whereas a random-effects model was employed when heterogeneity was substantial (I² > 50%) [23]. *P*-value < 0.05 was considered statistically significant.

### Quality assessment

The Jadad five-item scale was used to assess study quality [24]. We assigned a score ranging from 0 to 5 for each following items: randomization, double blinding and withdrawals. All studies included in our analysis had a score ≥ 3 (Supplementary Table 2).

### Risk of Bias assessment

The Cochrane Risk of Bias 2 Tool (RoB2) was used to assess the quality of trials included. According to Grading of Recommendations Assessment, Development, and Evaluation (GRADE), we categorized as “very low,” “low,” “moderate,” or “high” each of six domains including: randomization, allocation concealment, blinding of participants and personnel, blinding of outcome assessment, incomplete outcome data and selective reporting bias [25, 26].

## Results

### Characteristics of studies

**Fig. 1:**
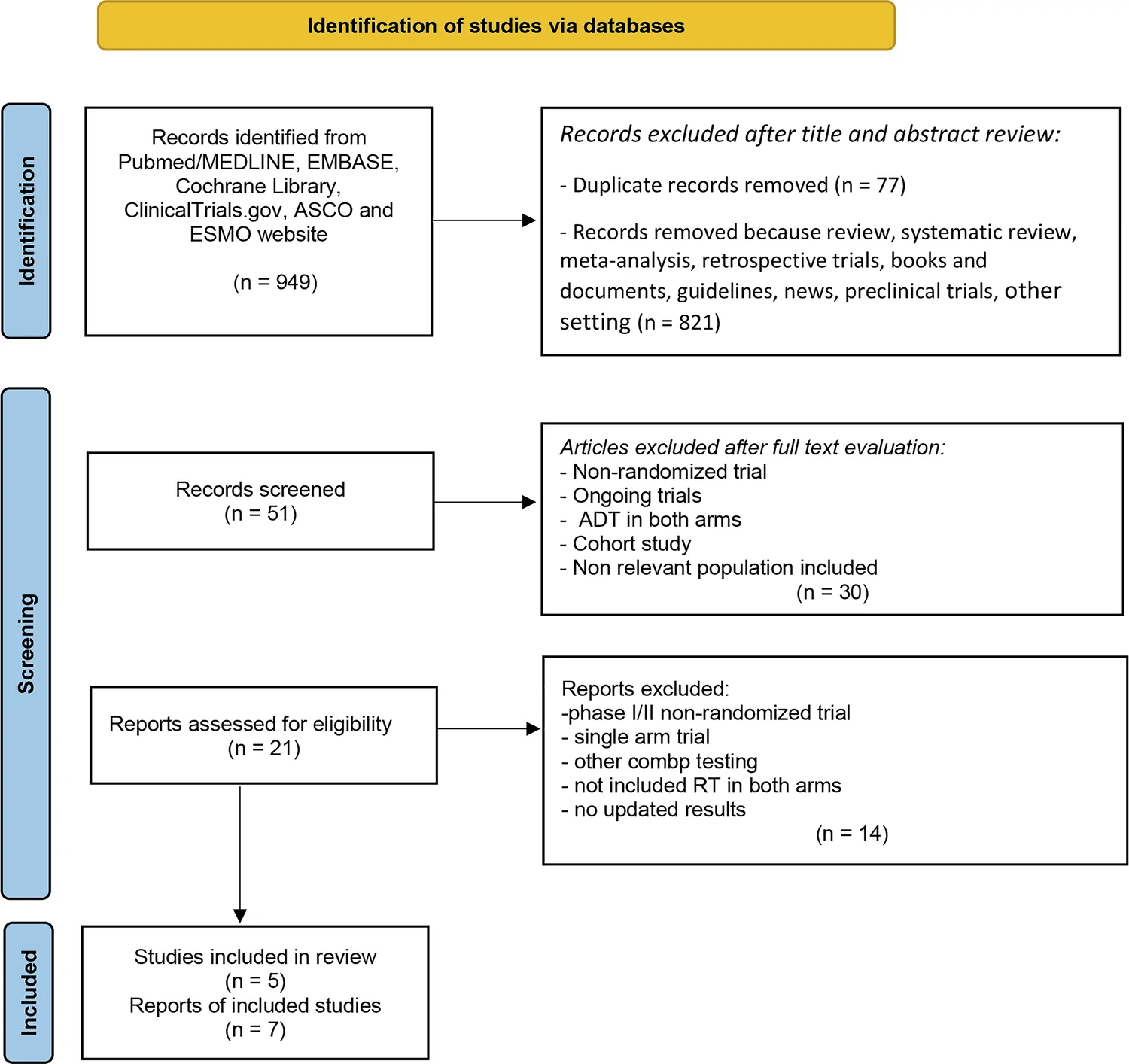
PRISMA Flowchart illustrating the study identification, screening, eligibility assessment, and inclusion process for the systematic review and meta-analysis.

A total of 949 records were included in the selection. After removal of duplicates and review, systematic review, meta-analysis, retrospective trials, guidelines, news, preclinical trials (821 records), 51 documents were screened for this meta-analysis. After eligibility check, a total of five RCTs (GETUG-AFU 16, RADICALS-HD, RTOG 0534 SPPORT, RTOG 9601 and SALV-ENZA) and seven records were selected for the analysis [11–17]. All the included studies were randomized phase II or III trials. A total of 4536 patients were included in the final analysis. The process of literature search and screening are detailed in Fig. 1.

### Metastasis free survival

**Fig. 2:**
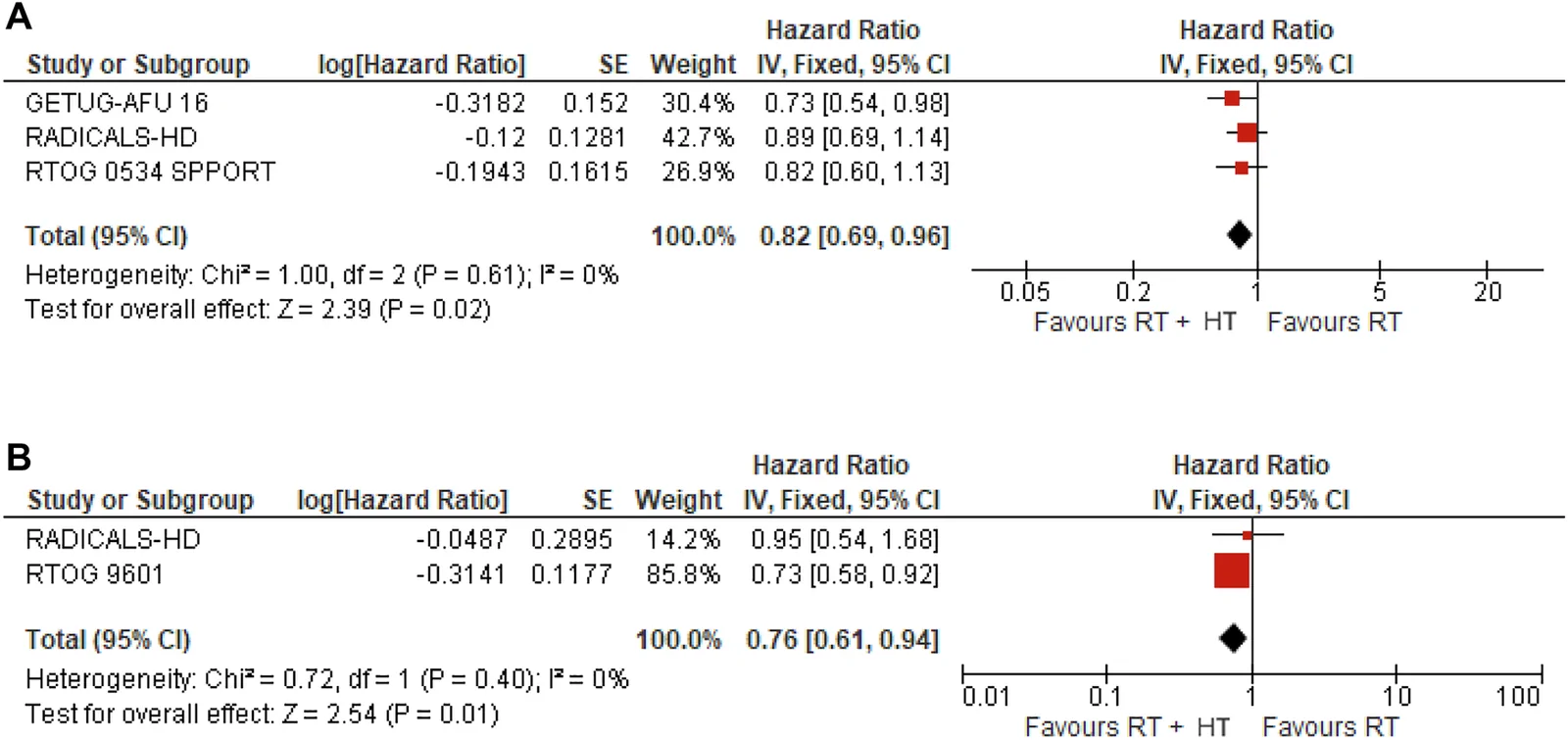
Effect of radiotherapy plus hormone therapy on metastasis-free survival. **A** Assessment of metastasis free survival (MFS) in patients treated with RT + HT short term vs. RT alone. **B** Assessment of MFS in patients treated with RT + HT long term vs. RT alone. Values < 1 favor the combination of RT plus HT.

**Fig. 3:**
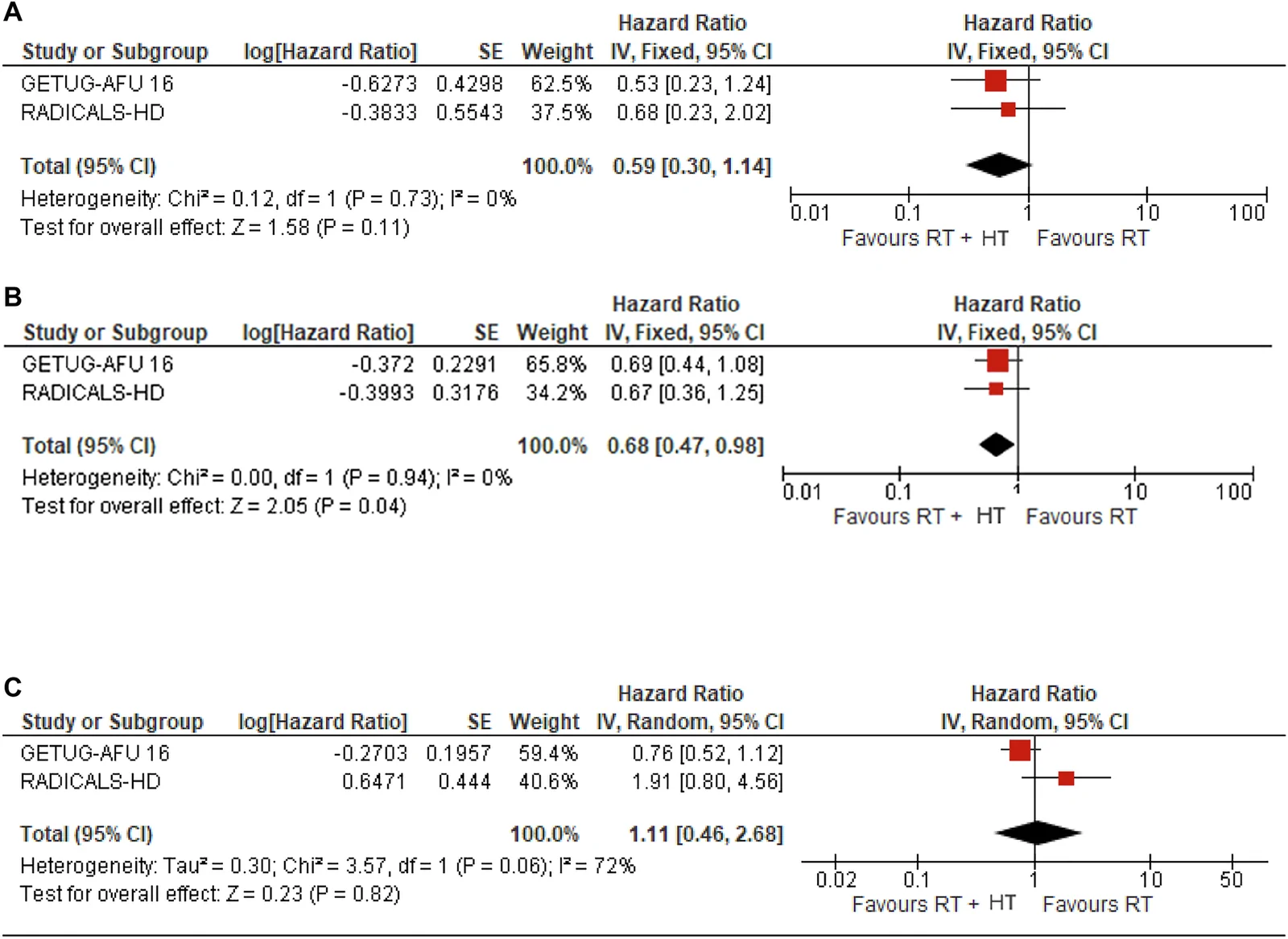
Subgroup analyses of metastasis-free survival according to pathological characteristics. **A** Assessment of MFS in patients with GS ≥ 8 treated with RT + short-term HT vs. RT alone. **B** Assessment of MFS in patients with positive margins treated with RT + HT vs. RT alone. **C** Assessment of MFS in patients with negative margins treated with RT + HT vs. RT alone.

Our analysis evaluated the benefit, in terms of MFS, of adding short-course HT (6 months) to SRT. Three studies were included in the final analysis (GETUG-AFU 16, RADICALS-HD and RTOG 0534 SPPORT). A total of 3364 patients were included. Short-term HT combined with SRT demonstrated to prolong MFS (fixed-effect; HR = 0.82; 95% CI: 0.69–0.96, *p* = 0.02). For the analysis of long-course HT (24 months) in combination with SRT, we considered two studies (RADICALS-HD and RTOG 9601). A total of 1086 patients were considered. Our findings confirmed a benefit also combining long- course HT with SRT (fixed effect; HR = 0.76; 95% CI: 0.61–0.94, *p* = 0.01). Subgroup analysis revealed a statistically significant benefit in MFS only in patients with positive surgical margins (fixed effect; HR = 0.68; 95% CI: 0.47–0.98, *p* = 0.04). In contrast, no significant improvement in MFS was observed in patients with Gleason score ≥ 8 (fixed effect; HR = 0.59; 95% CI: 0.30- 1.14, *p* = 0.11) or in patients with negative margins after RP (fixed effect; HR = 1.11; 95% CI: 0.46–2.68, *p* = 0.06) (Fig. 2 and Fig. 3).

### Progression free survival

**Fig. 4:**
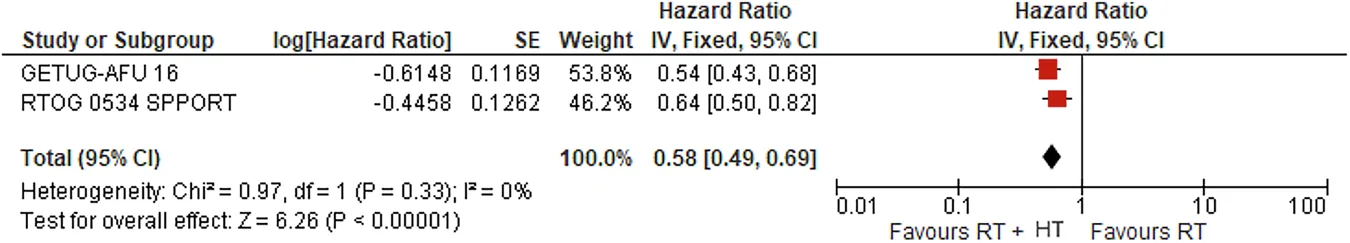
Assessment of progression free survival (PFS) in patients treated with RT + HT short term vs. RT alone.

For PFS analysis, data were available from two studies (GETUG-AFU 16 and RTOG 0534 SPPORT) assessing short course HT (for 6 months) in combination with SRT. A total of 1884 patients were considered. The analysis demonstrated that short-term HT combined with SRT improves PFS (fixed-effect; HR = 0.58; 95% CI: 0.49–0.69, *p* < 0.00001). Owing to insufficient data, it was not possible to perform the analysis for the subgroup treated with long-course HT (Fig. 4).

### Overall survival

**Fig. 5:**
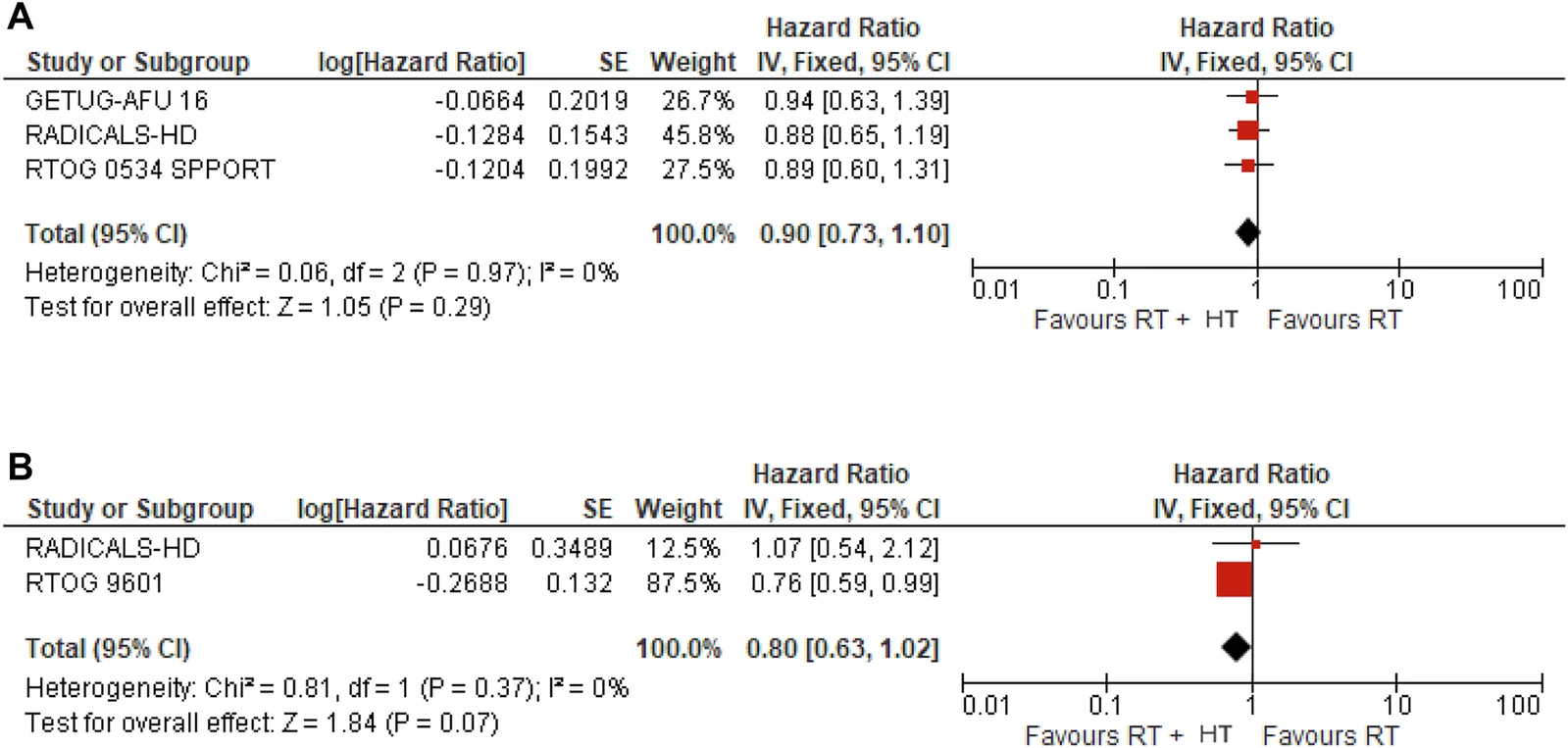
Effect of radiotherapy plus hormone therapy on overall survival. **A** Assessment of overall survival (OS) in patients treated with RT + HT short term vs. RT alone. **B** Assessment of OS in patients treated with RT + HT long term vs. RT alone.

To assess the OS benefit, data were extracted from three studies (GETUG-AFU 16, RADICALS-HD and RTOG 0534 SPPORT). A total of 3364 patients were considered. The pooled HR showed no OS benefit when comparing short-term HT with SRT versus SRT alone (fixed effect; HR = 0.90; 95% CI: 0.73–1.10, *p* = 0.30). For the analysis of long-course HT (24 months) in combination with SRT, data were available from two studies (RADICALS-HD and RTOG 9601). A total of 1086 patients were considered. The analysis confirmed no OS benefit, even with the addition of long-course HT (24 months) to SRT (fixed effect HR = 0.80; 95% CI: 0.63–1.02, *p* = 0.07)(Fig. 5).

### Biochemical recurrence free survival

**Fig. 6:**
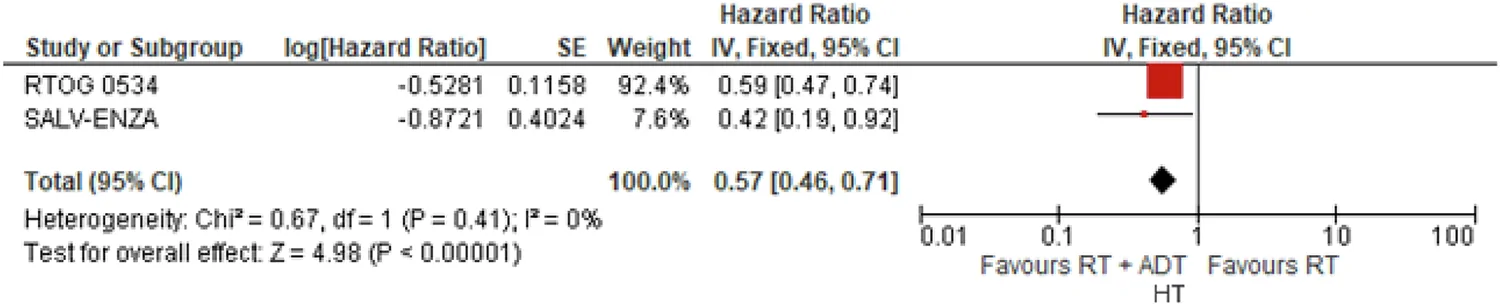
Assessment of biochemical progression free survival (bPFS) in patients treated with RT + HT short term vs. RT alone.

To evaluate the bPFS benefit, we considered data from two studies in the final analysis (RTOG 0534 and SALV-ENZA). A total of 1228 patients were included. The final analysis demonstrated improved bPFS when short-term HT was combined with SRT (fixed-effect; HR = 0.57; 95% CI: 0.46–0.71, *p* < 0.00001). Owing to insufficient data, it was not possible to perform the analysis for the subgroup treated with long-course HT (Fig. 6).

## Discussion

BCR is an independent risk factor for the development of DM, PCSM, and to a lesser extent overall mortality (OM) [2]. Notably, a PSA doubling time (PSA-DT) < 12 months and pathological Gleason Score (pGS) ≥ 8 are the main prognostic factors for DM, PCSM, and OM in patients with BCR following RP [2]. According to EAU guidelines, patients with BCR after RP can be stratified into low-risk BCR (PSA-DT > 12 months and pGS <8) or high-risk

BCR (PSA-DT < 12 months or pGS 8–10) [3]. Therefore, optimizing the clinical management, particularly for patients with high-risk BCR, remains an unmet need. SRT to the prostate bed has long been the standard of care, yet the added value of HT implementation remains uncertain. EAU guidelines provide only weak recommendations and clinical practice varies widely [3]. Given the low certainty of available evidence, we conducted a meta-analysis to better identify patients who derive the greatest benefit from combining SRT with HT. Our analysis was restricted to men with confirmed BCR, excluding adjuvant and immediate salvage RT to reduce heterogeneity and focus on a well-defined clinical scenario. To our knowledge, this is the first meta-analysis incorporating data from the SALV-ENZA trial, the first randomized study evaluating the addition of an ARPI to SRT in this setting [17]. Our findings consistently showed that the addition of HT to SRT significantly improved PFS, bPFS, and MFS. Subgroup analyses indicated that the MFS benefit was primarily observed in patients with positive surgical margins, with no clear advantage in those with pGS ≥8 or negative margins. This finding should be interpreted cautiously, as high-grade disease was underrepresented across most trials and subgroup analyses were not powered to detect differential benefit in this population. Although patients with high-grade disease (GG ≥ 4) are generally expected to derive greater benefit from systemic intensification, this effect was not clearly observed in our analysis, likely due to the limited representation of this subgroup across the included trials. Conversely, patients with EAU low-risk BCR may derive limited absolute benefit from ADT intensification, supporting a more individualized and potentially de-escalated approach in this subgroup.

In addition, the lack of a statistically significant OS improvement may be influenced by factors such as crossover, subsequent treatments, heterogeneous follow-up and competing risks, highlighting the need to refine patient selection.

Several differences across trials may have contributed to the variability in outcomes. Definitions of BCR varied across trials, with some studies including patients with persistently detectable PSA or PSA levels up to 2 ng/mL after surgery. All RCTs enrolled men with pT2-pT3 disease, whereas RADICALS-HD uniquely included pT1 cases. The proportion of patients with GS ≥ 8 was generally underrepresented among the trials included in the analysis, with the only exception being SALV-ENZA, which enrolled the highest proportion of patients with GS ≥ 8 (45%). Radiotherapy fields were generally limited to the prostate bed, with only a minority receiving pelvic nodal irradiation (PNRT). Importantly, PNRT was analysed as a separate arm only in RTOG 0534, and this arm was excluded from our analysis. Additionally, patients with positive nodal status after RP were excluded from

GETUG-AFU 16 and RTOG 0534. Moreover, RADICALS-HD trial randomized patients to receive RT alone, RT with short-course ADT or RT plus long-course ADT. Patients enrolled could choose two-way randomization (no ADT vs. short-course ADT) or three-way randomization (no ADT vs. short-course ADT vs. long-course ADT). Two-way randomization was the preferred option, with 1150 participants choosing it compared to 492 choosing the three-way option. Of those who opted for two-way randomization, 571 patients were assigned in the no ADT arm, whilst 166 patients were allocated to the no ADT arm with three-way randomization. Thus, our analysis considered a total of 737 patients in the no ADT arm for the RADICALS-HD trial. These differences in eligibility criteria, treatment strategies and endpoint definitions likely contributed to heterogeneity in the reported outcomes.

Follow-up duration also varied substantially across trials, ranging from a few years to more than a decade in RTOG 9601, potentially influencing OS estimates. Regarding PFS definitions, they differed among studies: since RADICALS-HD defined PFS exclusively based on clinical progression, its PFS data were not included in our pooled analysis [11–17]. Another important limitation is that, despite the recognized prognostic impact of PSA-DT, none of the included trials reported subgroup analyses according to the high-risk definition proposed by the EAU (PSA-DT < 12 months). Available subgroup data are mainly restricted to pathological features, such as GS/ISUP grade or surgical margin status, with only limited information for ISUP 4 disease and no stratification by PSA kinetics. As a result, the magnitude of benefit from adding ADT or ARPIs to SRT in patients with high-risk BCR according to EAU criteria remains largely unexplored.

Nevertheless, our results are aligned with the DADSPORT meta-analysis reporting improved MFS and prostate-cancer specific survival (PCSS) with either short- or long- course ADT combined with postoperative RT [27]. Similarly, a recent network meta-analysis by Le QC et al. evaluated the impact of concomitant ADT and its duration in the postoperative setting [28]. Compared with these analyses, our study specifically focuses on patients with confirmed BCR after RP and incorporates recent evidence on treatment intensification, including ARPI-based strategies.

However, the potential benefits of treatment intensification must be carefully balanced against its toxicity profile. Indeed, long-term HT is associated with a broad spectrum of adverse effects, including sexual dysfunction, hot flashes, gynecomastia, fatigue, decreased bone mineral density, neurocognitive and psychological sequelae, and an overall decline in quality of life (QoL) [29].

Although a RCT is currently ongoing to define the optimal duration of ADT in combination with SRT (NCT05781217), a promising strategy to refine treatment selection is the integration of molecular biomarkers into clinical decision-making [30]. Several genomic classifiers, including Decipher, Prolaris, and Oncotype DX, as well as emerging multigene signatures, have demonstrated improved ability to predict recurrence after RP in patients with adverse pathological features [31–35]. Beyond their prognostic role, these genomic tools are increasingly being investigated as potential predictive biomarkers to identify patients more likely to benefit from treatment intensification strategies. Supporting this evidence, the first biomarker-driven RCT showed that patients with BCR and luminal B prostate cancer, identified using the PAM50 genomic signature, experienced a significant improvement in MFS and biochemical progression when apalutamide was added to SRT [36]. Similarly, the detection of circulating prostate cells (CPCs) after surgery has also been associated with an increased risk of early BCR and disease progression [37].

In addition to tissue-based biomarkers, advances in molecular imaging may further refine patient stratification at the time of BCR. In particular, PSMA PET has demonstrated superior sensitivity for detecting early metastatic disease and may help guide treatment intensification strategies [38]. Notably, a randomized phase II trial demonstrated that PSMA PET-guided SRT improved failure-free survival and delayed the need for subsequent therapies, with a greater benefit observed among patients who did not receive concomitant ADT with SRT [39].

In parallel, intensification of the radiotherapy field represents another potential strategy. This approach was supported by the RTOG 0534/SPPORT trial, in which the addition of PNRT combined with short-term ADT significantly improved PFS compared with prostate bed RT alone [14]. However, whether pelvic nodal irradiation can replace systemic therapy in biologically low-risk patients remains uncertain. Future trials incorporating molecular and imaging biomarkers will be crucial to define whether treatment escalation should preferentially rely on systemic intensification, radiation field expansion, or a tailored combination of both. In this rapidly evolving therapeutic landscape, our work reinforces the view that prostate cancer management should increasingly rely on the biological and clinical characteristics of each patient, ensuring a truly individualized approach.

Beyond radiotherapy-based intensification strategies, systemic approaches are also being explored in patients with biochemical recurrence. In the phase III EMBARK trial, patients with high-risk biochemical recurrence were randomized to enzalutamide plus ADT, ADT

alone, or enzalutamide monotherapy [40]. Enzalutamide plus ADT significantly improved metastasis-free and overall survival compared with ADT alone, whereas enzalutamide monotherapy improved metastasis-free survival without a significant overall survival benefit. Importantly, patients enrolled in EMBARK were not candidates for salvage radiotherapy, and therefore these findings reflect a different clinical scenario from the post- prostatectomy salvage setting evaluated in the present meta-analysis.

## Conclusion

Our meta-analysis indicates that combining hormonal therapy with salvage radiotherapy may improve biochemical and metastasis-related outcomes in selected patients with biochemical recurrence after radical prostatectomy. Nevertheless, the lack of a consistent overall survival benefit and the variability across trials highlight the importance of careful patient selection. Future research integrating molecular biomarkers and advanced imaging strategies will be crucial to refine risk stratification and guide individualized treatment intensification.

## Supplementary information

supplementary legends

Supplemental Material 1

Supplemental Material 2

Supplemental Material 1

Supplemental Material 2
