# Supplementary material for: Salvage radiotherapy with or without hormonal therapy for biochemical recurrence after radical prostatectomy: A systematic review and meta-analysis
Source: Prostate Cancer Prostatic Dis. 2026 May 18;29(5):951–7. doi: 10.1038/s41391-026-01120-9 (PMC13719526; doi:10.1038/s41391-026-01120-9)
Supplement: Supplementary file 1 [file 41391_2026_1120_MOESM2_ESM.docx]

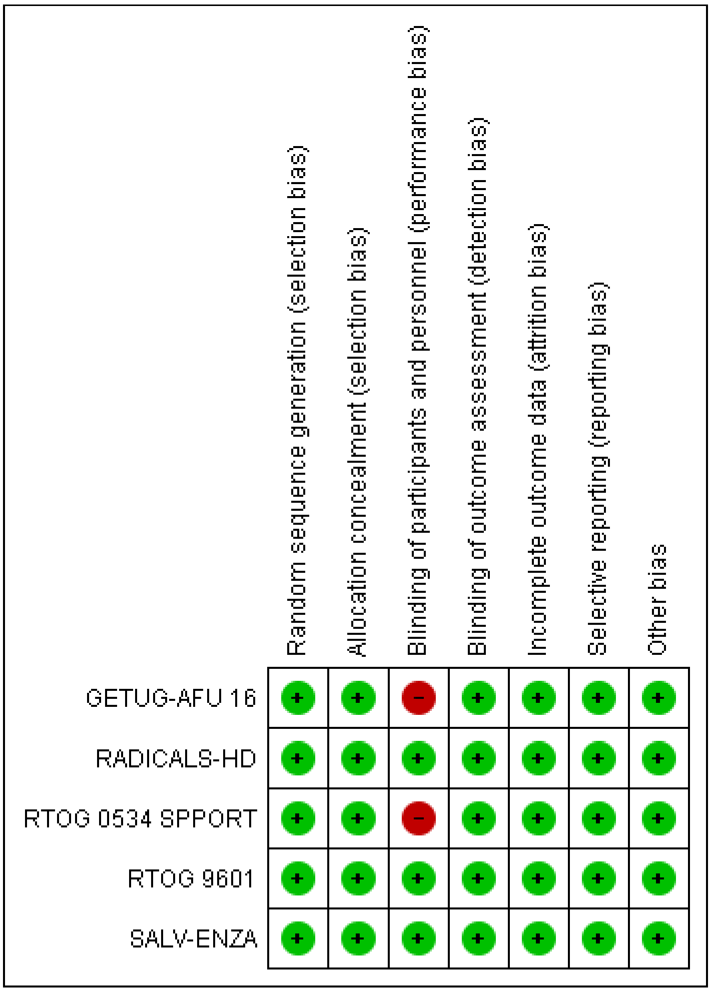
Random sequence generation (selection bias)
Allocation concealment (selection bias)
Blinding of participants and personnel (performance bias)
Blinding of outcome assessment (detection bias)
Incomplete outcome data (attrition bias)
Selective reporting (reporting bias)
Other bias
GETUG-AFU 16
RADICALS-HD
RTOG 0534 SPPORT
RTOG 9601
SALV-ENZA
