# Supplementary material for: Salvage radiotherapy with or without hormonal therapy for biochemical recurrence after radical prostatectomy: A systematic review and meta-analysis
Source: Prostate Cancer Prostatic Dis. 2026 May 18;29(5):951–7. doi: 10.1038/s41391-026-01120-9 (PMC13719526; doi:10.1038/s41391-026-01120-9)
Supplement: Supplementary file 2 [file 41391_2026_1120_MOESM3_ESM.docx]

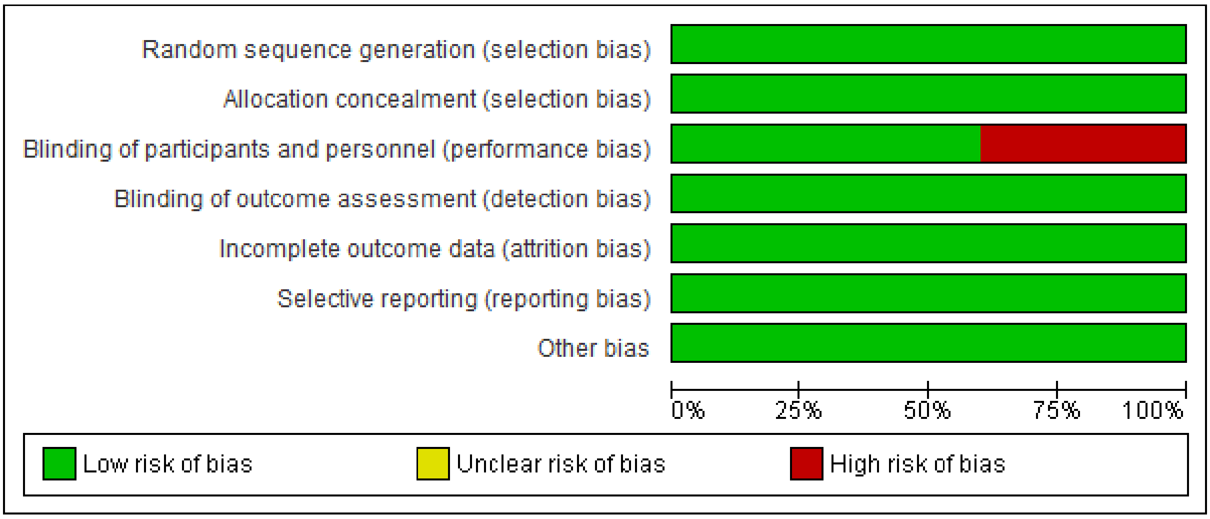
Random sequence generation (selection bias)
Allocation concealment (selection bias)
Blinding of participants and personnel (performance bias)
Blinding of outcome assessment (detection bias)
Incomplete outcome data (attrition bias)
Selective reporting (reporting bias)
Other bias
0%
25%
50%
75%
100%
Low risk of bias
Unclear risk of bias
High risk of bias
