# Supplementary material for: Salvage radiotherapy with or without hormonal therapy for biochemical recurrence after radical prostatectomy: A systematic review and meta-analysis
Source: Prostate Cancer Prostatic Dis. 2026 May 18;29(5):951–7. doi: 10.1038/s41391-026-01120-9 (PMC13719526; doi:10.1038/s41391-026-01120-9)
Supplement: Supplementary file 1 [file 41391_2026_1120_MOESM1_ESM.docx]

**Supplementary Figure 1.** Traffic light plot summarizing the risk of bias assessment for the five randomized phase II-III trials included in the meta-analysis (GETUG-AFU 16, RADICALS-HD, RTOG 0534/SPPORT, RTOG 9601, and SALV-ENZA), evaluated using the Cochrane Risk of Bias tool. Each domain is classified as low risk (green), high risk (red), or unclear risk (yellow). Domains assessed include random sequence generation, allocation concealment, blinding of participants and personnel, blinding of outcome assessment, incomplete outcome data, selective reporting, and other potential sources of bias.

**Supplementary Figure 2.** Bar chart summarizing the proportion of randomized trials classified as having low (green), unclear (yellow), or high (red) risk of bias for each methodological domain, according to the Cochrane Risk of Bias tool. Overall, the included trials showed a low risk of bias across most domains. A higher risk was observed for blinding of participants and personnel, reflecting the open-label design of some studies, while all other domains, including random sequence generation, allocation concealment, outcome assessment, incomplete outcome data, and selective reporting-were predominantly rated as low risk.

**Supplementary Table 1**. 27 item PRISMA Checklist 2020.

**Supplementary Table 2.** Selected randomized phase II and III trials for final analysis.

1. Patients who received nodal pelvic irradiation: 56 (15%) in the radiotherapy alone group and 63 (17%) in the radiotherapy plus goserelin group.
2. Patients randomly assigned to group 3 received PLNRT plus PBRT with short-term ADT. 3. Patients who received nodal pelvic irradiation: 37 (5%) in the radiotherapy alone group and 51 (7%) in the radiotherapy plus 6mo ADT group.

4. Patients who received nodal pelvic irradiation: 13 (8%) in the radiotherapy alone group, 11 (7%) in the radiotherapy plus 6mo ADT group and 9 (6%) in the radiotherapy plus 24mo ADT group. RT: radiotherapy; PBRT: prostate bed radiotherapy; PLNRT: pelvic nodes radiotherapy; ADT: androgen deprivation therapy; PBO: placebo; enza: enzalutamide: PFS: progression free survival; OS: overall survival; MFS: metastasis free survival; BPFS: biochemical progression free survival; mo: months; NA: not available; HR: hazard ratio.
