# Supplementary material for: Salvage radiotherapy with or without hormonal therapy for biochemical recurrence after radical prostatectomy: A systematic review and meta-analysis
Source: Prostate Cancer Prostatic Dis. 2026 May 18;29(5):951–7. doi: 10.1038/s41391-026-01120-9 (PMC13719526; doi:10.1038/s41391-026-01120-9)
Supplement: Supplementary file 5 [file 41391_2026_1120_MOESM5_ESM.docx]

| **Trial** | **Phase** | **Trial Design** | | | | | | **Duration**  **of ADT** | **Pelvic irradiation**  **allowed**  **(yes/no)** | **Median follow-up** | **PFS**  **(PBRT + 6 mo ADT/RT)**  **(HR, 95%CI; p value)** | **OS**  **(PBRT + 6mo ADT / RT)**  **(HR, 95%CI;**  **p value)** | **MFS**  **(PBRT + 6 mo ADT / RT)**  **(HR, 95%CI;**  **p value)** | **BPFS**  **(PBRT + 6 mo ADT / RT)**  **(HR, 95%CI;**  **p value)** | **Jadad**  **score** |
| --- | --- | --- | --- | --- | --- | --- | --- | --- | --- | --- | --- | --- | --- | --- | --- |
| GETUG-AFU 16 | III | RT | 373 | RT +  ADT | 369 | \ | \ | 6 mo | Yes^1^ | 112 mo | HR 0.54  (0.43,0.68); p<0.0001 | HR 0.93;  (0.63,1.39); p=0.73 | HR 0.73  (0.54,0.98);  p=0.0339 | NA | 3 |
| RTOG 0534 SPPORT | III | PBRT | 564 | PBRT + ADT | 578 | PBRT + PLNRT + ADT | 574 | 4-6 mo | Yes² | 98 mo | HR 0.64  (0.50,0.82); p <0.0001 | HR 0.89  (0.60,1.31);  p= 0.245 | HR 0.82  (0.60,1.13);  p= 0.082 | HR 0.59  (0.47,0.74); p<0.0001 | 3 |
| SALV-ENZA | II | RT +  PBO | 43 | RT +  enza | 43 | \ | \ | 6 mo | No | 34 mo | NA | NA | NA | HR 0.42  (0.19, 0.92);  p = 0.031 | 3 |
| RADICALS-HD  (2-way  randomisation) | III | RT | 737 | RT +  6mo ADT | 743 | \ | \ | 6 mo | Yes^3^ | 108 mo | NA | HR 0.882  (0.651,1.194) p=0.42 | HR 0.886  (0.688,1.140);  p= 0.35 | NA | 3 |
| Trial | **Phase** | **Trial Design** | | | | | | **Duration**  **of ADT** | **Pelvic irradiation**  **allowed**  **(yes/no)** | **Median follow-up** | **PFS**  **(PBRT + 24 mo ADT/RT)**  **(HR, 95%CI; p value)** | **OS**  **(PBRT + 24mo ADT / RT)**  **(HR, 95%CI;**  **p value)** | **MFS**  **(PBRT + 24mo ADT / RT)**  **(HR, 95%CI;**  **p value)** | **BPFS**  **(PBRT + 24mo ADT / RT)**  **(HR, 95%CI;**  **p value)** | **Jadad**  **score** |
| RADICALS-HD  (3-way randomisation) | III | RT | 164 | RT + 6mo ADT | 164 | RT + 24mo ADT | 162 | 6/24 mo | Yes^4^ | 108 mo | NA | HR 1.07  (0.54, 2.12)  p = 0.940 | HR 0.95  (0.54, 1.68)  p = 0.43 | NA | 3 |
| RTOG 9601 | III | RT + placebo | 376 | \ | \ | RT + 24mo ADT | 384 | 24 mo | No | 156 mo | NA | HR 0.77  (0.59,0.99);  p= 0.04 | HR 0.73  (0.58, 0.92)  p= 0.006) | HR 0.48  (0.40, 0.59);  p < 0.001 | 3 |
